# Transorbital approach to the cavernous sinus: an anatomical study of the related cranial nerves

**DOI:** 10.3389/fnana.2024.1367533

**Published:** 2024-04-17

**Authors:** Alejandra Mosteiro, Marta Codes, Roberto Tafuto, Roberto Manfrellotti, Jorge Torales, Joaquim Enseñat, Alberto Di Somma, Alberto Prats-Galino

**Affiliations:** ^1^Laboratory of Surgical Neuroanatomy (LSNA), Faculty of Medicine and Health Sciences, Universitat de Barcelona, Barcelona, Spain; ^2^Department of Neurological Surgery, Hospital Clínic de Barcelona, Barcelona, Spain; ^3^Division of Neurosurgery, Department of Neurosciences, Reproductive and Odontostomatological Sciences, Università Degli Studi di Napoli “Federico II”, Naples, Italy; ^4^Division of Neurosurgery, Azienda Socio Sanitaria Territoriale - Monza, Ospedale San Gerardo, Monza, Italy; ^5^Institut d’Investigacions Biomèdiques August Pi i Sunyer (IDIBAPS), Barcelona, Spain

**Keywords:** cavernous sinus, transorbital, endoscopic, superior eyelid, cranial nerve, interdural peeling

## Abstract

**Background:**

The cavernous sinus (CS) is a demanding surgical territory, given its deep location and the involvement of multiple neurovascular structures. Subjected to recurrent discussion on the optimal surgical access, the endoscopic transorbital approach has been recently proposed as a feasible route for selected lesions in the lateral CS. Still, for this technique to safely evolve and consolidate, a comprehensive anatomical description of involved cranial nerves, dural ligaments, and arterial relations is needed.

**Objective:**

Detailed anatomical description of the CS, the course of III, IV, VI, and V cranial nerves, and C3-C7 segments of the carotid artery, all described from the ventrolateral endoscopic transorbital perspective.

**Methods:**

Five embalmed human cadaveric heads (10 sides) were dissected. An endoscopic transorbital approach with lateral orbital rim removal, anterior clinoidectomy, and petrosectomy was performed. The course of the upper cranial nerves was followed from their apparent origin in the brainstem, through the middle fossa or cavernous sinus, and up to their entrance to the orbit. Neuronavigation was used to follow the course of the nerves and to measure their length of surgical exposure.

**Results:**

The transorbital approach allowed us to visualize the lateral wall of the CS, with cranial nerves III, IV, V1-3, and VI. Anterior clinoidectomy and opening of the frontal dura and the oculomotor triangle revealed the complete course of the III nerve, an average of 37 (±2) mm in length. Opening the trigeminal pore and cutting the tentorium permitted to follow the IV nerve from its course around the cerebral peduncle up to the orbit, an average of 54 (±4) mm. Opening the infratrochlear triangle revealed the VI nerve intracavernously and under Gruber’s ligament, and the extended petrosectomy allowed us to see its cisternal portion (27 ± 6 mm). The trigeminal root was completely visible and so were its three branches (46 ± 2, 34 ± 3, and 31 ± 1 mm, respectively).

**Conclusion:**

Comprehensive anatomic knowledge and extensive surgical expertise are required when addressing the CS. The transorbital corridor exposes most of the cisternal and the complete cavernous course of involved cranial nerves. This anatomical article helps understanding relations of neural, vascular, and dural structures involved in the CS approach, essential to culminating the learning process of transorbital surgery.

## 1 Introduction

The cavernous sinus (CS) is a beautiful, delicate, and complex venous entity within the sphenoidal and temporal skull base, comprising multiple neurovascular relations. Indeed, the composite anatomy of the CS and the proximity to crucial neural and vascular structures have generated great controversy over the optimal surgical approach to this area, with many lesions considered unresectable and prone to non-surgical management (Fariselli et al., 2016; Corniola et al., 2022). Even when conventional transcranial approaches are, thus far, the gold standard (Dolenc, 1985; Al-Mefty and Anand, 1990; Dolenc, 1997; Heth and Al-Mefty, 2003; Matsuo et al., 2016), the direct pathway afforded by ventral routes like the transorbital (TO) and the minimally disruptive manipulation granted by the endoscopic techniques have rekindled surgical attention to this area (Cappabianca et al., 1998; de Divitiis, 2003; Cavallo et al., 2005; Doglietto et al., 2009; Altay et al., 2012; Bly et al., 2014; Dallan et al., 2017; Di Somma et al., 2018b; Zoia et al., 2023). Still, it is only through an extensive anatomical expertise that this region becomes a scrutable surgical site.

This anatomical article provides a step-by-step explanation of how to approach the CS through the superior eyelid TO approach. Emphasis is made on key anatomical references, and how to perform the interdural and extradural peeling of the CS. The cranial nerves related to the CS are delineated, from their apparent origin in the brainstem to their course in the middle fossa or CS, up to their entrance in the orbit at the annulus of Zinn. Numerical data are provided on the length of their course seen from the TO perspective.

## 2 Methods

### 2.1 Specimens and materials

Ethical approval was obtained from the IRB of the University of Barcelona (Barcelona, Spain). All the dissections were performed at the Laboratory of Surgical NeuroAnatomy (LSNA) at the University of Barcelona. Five human specimens (10 sides) fixated with Cambridge solution were used, none of which had previously known neurological diseases. All specimens underwent a basal magnetic resonance imaging (MRI), with T1, T2, and diffusion-weighted (DTI) sequences, and a computed tomography (CT) scan, with 0.5 mm axial slices and 0° gantry angle. Six screws were implanted before CT scanning and used as fiducials for neuro-navigation (Brainlab, Germany).

Procedures were performed with a 4-mm diameter rigid endoscope, 18-cm long, 0° optics (Karl Storz), connected to a light source through a fiber-optic cable (300WXenon, Karl Storz). A HD 4 K camera was used (Endovision Telecam SL; Karl Storz).

### 2.2 Quantitative data analysis

The course of each of the targeted cranial nerves was followed from proximal to distal by co-registering the MRI with the neuro-navigation system. Points were sequentially obtained, with a systematic separation of 5 mm, from the closest point to their apparent origin at the brainstem as seen from the TO perspective, till their exit toward the infratemporal fossa (V3), the pterygopalatine fossa (V2), or the orbit (VI, V1, IV, and III). The points thus obtained were transferred to the Brainlab workstation, and the complete length of each nerve was obtained by adding up the distance between the points. Quantitative data are expressed by means (± standard deviation).

### 2.3 Stepwise surgical technique

#### 2.3.1 Skin phase and working space

The skin incision is made in the superior eyelid crease to minimize cosmetic defects, extending from the supraorbital notch to the level of the lateral orbital canthus; at this point, the incision is curved laterally, to prevent it from coursing over the lateral canthal tendon. Beneath the skin, the fibers of the orbicularis oculi muscle are identified and divided; care should be taken not to damage the subjacent pretarsal fascia, i.e., a white plane connecting the septum and the levator palpebrae muscle. This avascular plane is followed superiorly until the orbital rim and the frontal process of the zygoma are recognized. Traction stitches are placed to keep the superior eyelid incision open.

The periosteum is cut over the orbital rim and dissected toward the orbit, while preserving the periorbital fascia for close-up reconstruction at the end of the surgery. Dynamic retraction is applied to displace the orbit medially so the endoscope can be inserted at the superolateral corner of the corridor. At this point, the inferior (IOF) and superior (SOF) orbital fissures are the two anatomical landmarks for beginning the orbital craniectomy.

#### 2.3.2 Bone phase

The lateral wall of the orbit extending between the IOF and SOF is mainly comprised of the greater sphenoid wing (GSW) and part of the zygomatic body. Drilling of the GSW will expose the temporalis muscle, and deeper into the corridor, the temporal dura. The floor of the middle cranial fossa should be flattened (Guizzardi et al., 2022) and the sagittal crest removed (Corrivetti et al., 2022), particularly its last piece corresponding to the crista ovale (Yanez-Siller et al., 2022). This will reveal the foramen rotundum with V2. Identification of V2 is essential for the interdural peeling of the CS.

Bone drilling continues toward the lesser sphenoid wing (LSW), revealing the frontal dura and the meningo-orbital band (MOB). The anterior clinoid process is then identified and freed from its dural attachments. An anterior clinoidectomy is performed, by egg-shell drilling of the clinoid and subsequent *en bloc* removal.

The lateral orbital rim (LOR) may be removed before or after the anterior clinoidectomy, to increase the surgical maneuverability and the visualization of the clinoidal and opticocarotid region. Removing the LOR with the cutting drill is performed with two sequential cuts: a horizontal cut from the IOF toward the opened temporal fossa and a vertical cut from the IOF upward and up to 5–10 mm above the frontal-zygomatic suture.

#### 2.3.3 Dura phase: interdural and extradural peeling

Once the bone work is accomplished, the interdural and extradural planes need to be identified and opened. The extradural plane extends between the temporal dura and the bony floor of the middle cranial fossa. The interdural plane extends between the dura propria of the temporal lobe and the dura of the lateral wall of the CS.

The extradural peeling is performed by lifting the temporal lobe from its dural attachments to the middle fossa floor. The middle meningeal artery (MMA) is encountered and cut. The interdural peeling starts at the level of the foramen rotundum/V2 (Dolenc, 1983; Perneczky et al., 1988; Yaşargil, 1996). The MOB cut is advisable to facilitate the mobilization of the temporal dura laterally (Dallan et al., 2017). The two planes of dissection (inter and extradural) join at the level of V3. Further dissection will reveal the Gasserian ganglion (Meckel’s cave), the petrous apex, the petrous ridge, and the tentorial insertion.

#### 2.3.4 Anterior petrosectomy and opening of the tentorium

The landmarks for anterior petrosectomy are identified, such as the V3 branch, the greater superficial petrosal nerve (GSPN) marking the petrous segment of the internal carotid (pICA), the petrous ridge, and the arcuate eminence. Drilling of the petrous apex will reveal the dura of the posterior fossa, which can be opened at this point. Care should be taken not to damage the 7–8th cranial nerve complex and not to extend the petrosectomy laterally to the level of the internal acoustic meatus.

After the petrosectomy, the tentorium can be detached from its insertion at the petrous ridge and cut up to the tentorial incisura. This increases the visualization of the pons and cerebral peduncles. Opening the tentorium right above or lateral to the trigeminal pore, but not medially, prevents damaging the IV cranial nerve.

See Supplementary Video 1 for a stepwise dynamic illustration.

## 3 Results

### 3.1 Interdural and extradural peeling of the cavernous sinus

**Figure 1:**
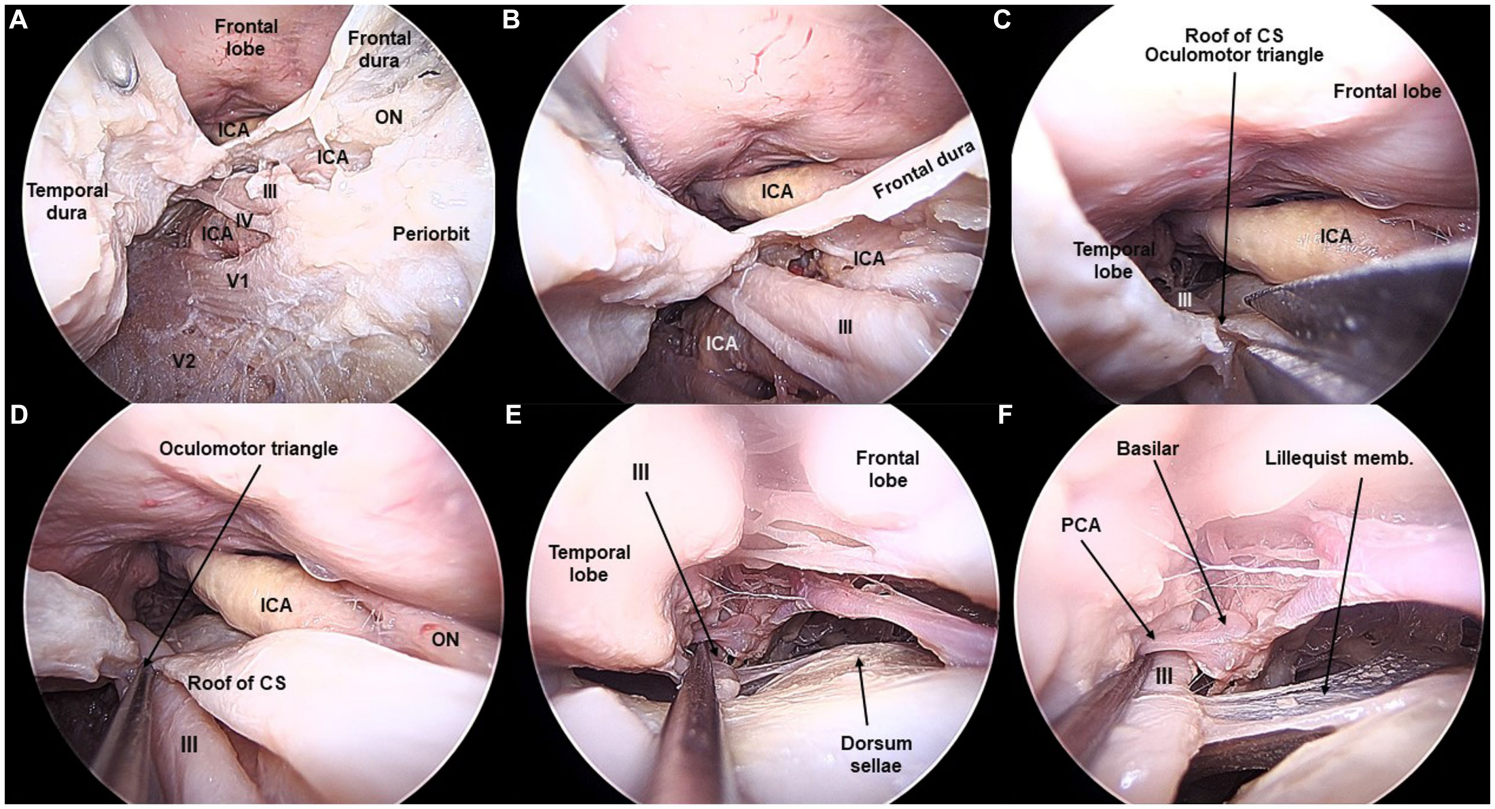
Lateral wall of the cavernous sinus and third cranial nerve exposed from the transorbital perspective (Cadaveric dissection, left orbit). **(A)** The interdural peeling unveils the lateral wall of the cavernous sinus (CS). A thin dural layer allows to observe the cranial nerves coursing in the lateral wall. Opening of the dura of the frontal lobe and opening the infratrochlear triangle allows to visualize the cavernous, clinoidal, ophthalmic, and communicating segments of the internal carotid artery (ICA). *ON, Optic Nerve*. **(B)** After the anterior clinoidectomy, the III cranial nerve is seen lateral and underneath the clinoidal segment of the ICA. **(C)** In the roof of the CS, the oculomotor triangle is opened, so that **(D)** the course of the III cranial nerve is fully seen. **(E,F)** The III nerve is followed in the posterior fossa towards the interpeduncular cistern. The basilar artery and the posterior cerebral artery are shown in relation to the III nerve.

The lateral wall of the CS is of utmost importance in the TO approach, becoming the main surgical reference. It is composed of a two-layer dura fold, which allows to perform an interdural peeling that unveils the CS without transgression. At the foramen rotundum, V2 marks the point where the junction of these two dura layers is naturally distinguished. A sharp division with a dissector directed upward, will separate the two layers aside. The temporal lobe, protected by the meningeal layer, is freed from the dura attachments, and laterally displaced as the interdural dissection moves superiorly and posteriorly, revealing the lateral wall of the CS protected by the endosteal dura layer. The cranial nerves III, IV, V1 and V2 become apparent in the lateral wall of the CS (Figure 1A). The VI nerve is not yet seen.

The extradural peeling separates the dura propria of the temporal lobe from the inferior margin of the CS and the middle fossa floor. The dissection begins at the inferomedial surface of the temporal lobe, just where the crista ovale lay before its removal. The floor of the middle fossa is exposed epidurally by lifting the temporal lobe dura with a dissector. Dissection continues until the foramen spinosum with the MMA is encountered laterally, the foramen rotundum/V2 medially and the foramen ovale/V3 posteriorly. After cutting the MMA, the epidural plane can be followed further posteriorly until the petrous ridge and apex are reached. Cutting off the dura connections extending between V2 and V3 will communicate the interdural and extradural spaces, thus increasing the exposure and maneuverability in the lateral cavernous region.

### 3.2 The cavernous sinus seen from the transorbital perspective

**Figure 2:**
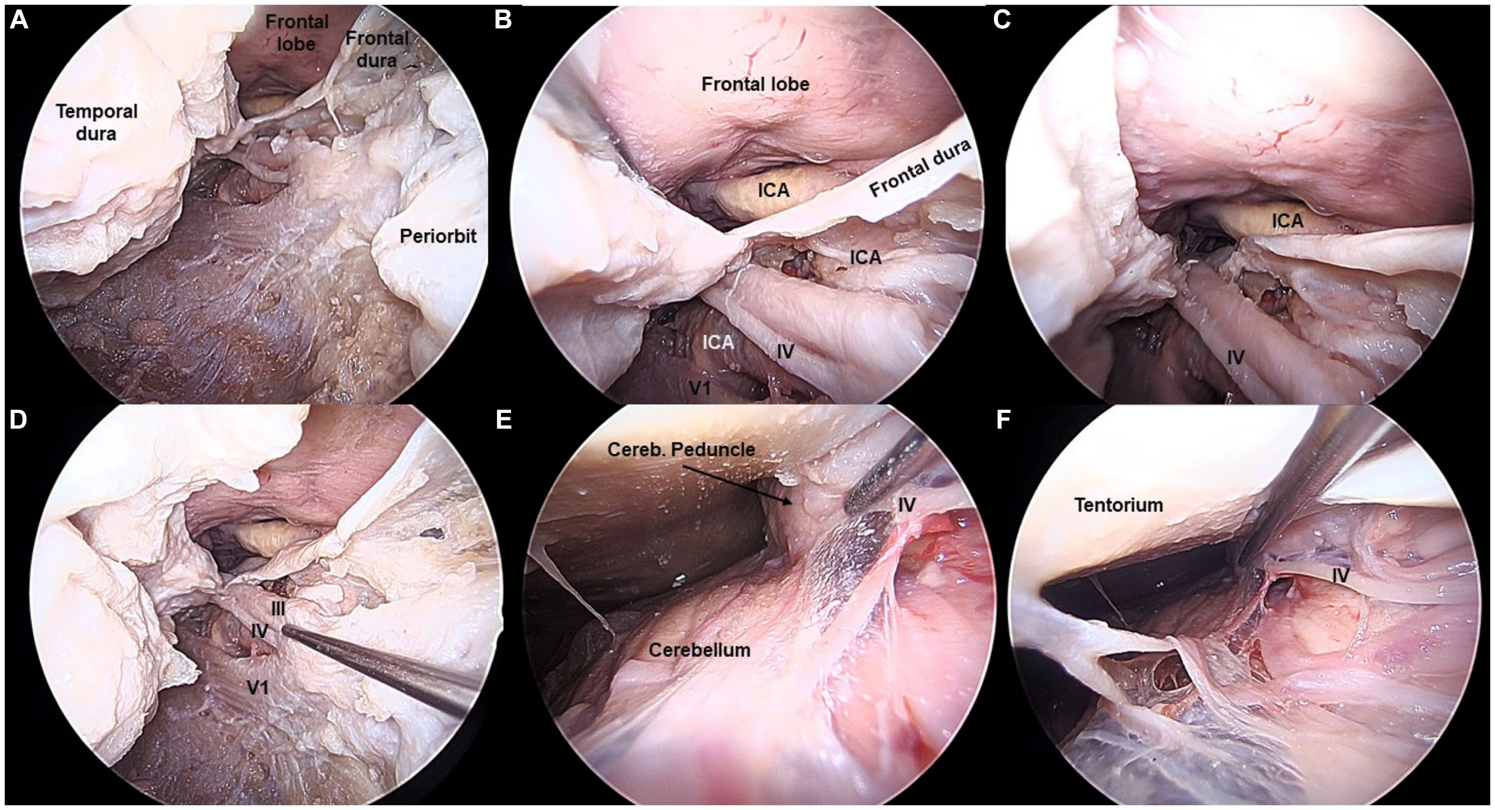
Fourth cranial nerve exposed from the transorbital perspective (Cadaveric dissection, left orbit). **(A,B)** The IV cranial nerve is exposed in the lateral wall of the cavernous sinus (CS), right beneath the III nerve. **(C,D)** The IV nerve is followed from the CS towards the posterior fossa, by opening the oculomotor triangle. **(E,F)** The IV nerve is shown in its cisternal segment, as it courses around the cerebellar peduncle. The apparent origin is not visible from the transorbital perspective.

For anatomical purposes, an exploration of the contents of the inner CS was performed. Opening the interdural plane reveals the lateral wall of the CS protected by the thin inner dura layer. The cranial nerves that run in this lateral wall are seen, from superior to inferior: the oculomotor (III) and trochlear (IV) nerves, and the ophthalmic (V1) and maxillary (V2) divisions of the trigeminal nerve. V3 does not pertain to the lateral wall of the CS; it rather runs in the middle fossa floor toward the foramen ovale and infratemporal fossa. Removing the anterior clinoid completely exposes the III nerve and its relationship with the clinoidal segment of the ICA. Both the proximal and distal dural rings are seen as the ICA becomes intradural (Figures 1, 2).

**Figure 3:**
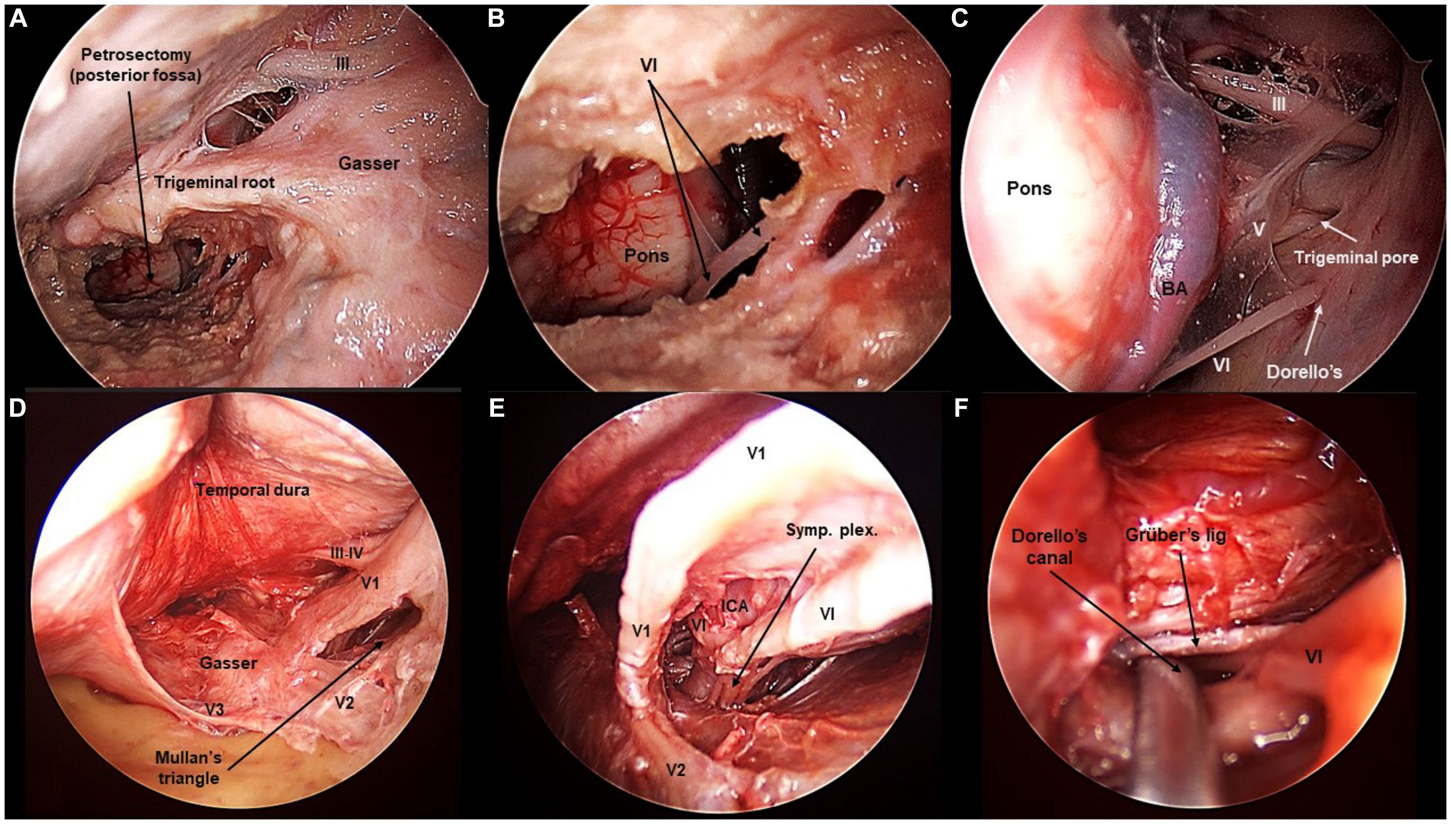
Sixth cranial nerve exposed from the transorbital perspective (Cadaveric dissection, left orbit). **(A,B)** The extended anterior petrosectomy, achieved after removal of the lateral orbital rim, reveals the VI cranial nerve in the posterior fossa. **(C)** The extended petrosectomy allows to visualize the retroclival area from the transorbital view. The contralateral VI nerve is seen, entering Dorello’s canal. Additionally, the V contralateral cranial nerve is seen entering the trigeminal pore, and so it is the contralateral III nerve entering the oculomotor triangle. **(D)** In the middle fossa, Mullan’s triangle is opened. **(E)** Opening Mullan’s triangle reveals the cavernous segment of the internal carotid artery (ICA) with the sympathetic plexus. The IV nerve is seen in intimal relation to the ICA. **(F)** The IV nerve can be followed intracavernously until its entrance through Dorello’s canal.

Parkinson’s (infratrochlear) triangle is delimited by the IV and V1 nerves. Mullan’s anteromedial triangle is limited by V1 and V2. Opening Parkinson’s triangle affords direct access to the posterior bend of the cavernous segment of the ICA (Corrivetti et al., 2023). Opening Mullan’s triangle by gentle retraction of V1 exposes the abducens (VI) nerve and, more deeply, also the ICA accompanied by the sympathetic plexus (Figure 3).

### 3.3 Cranial nerve course and length of surgical exposure

**Figure 4:**
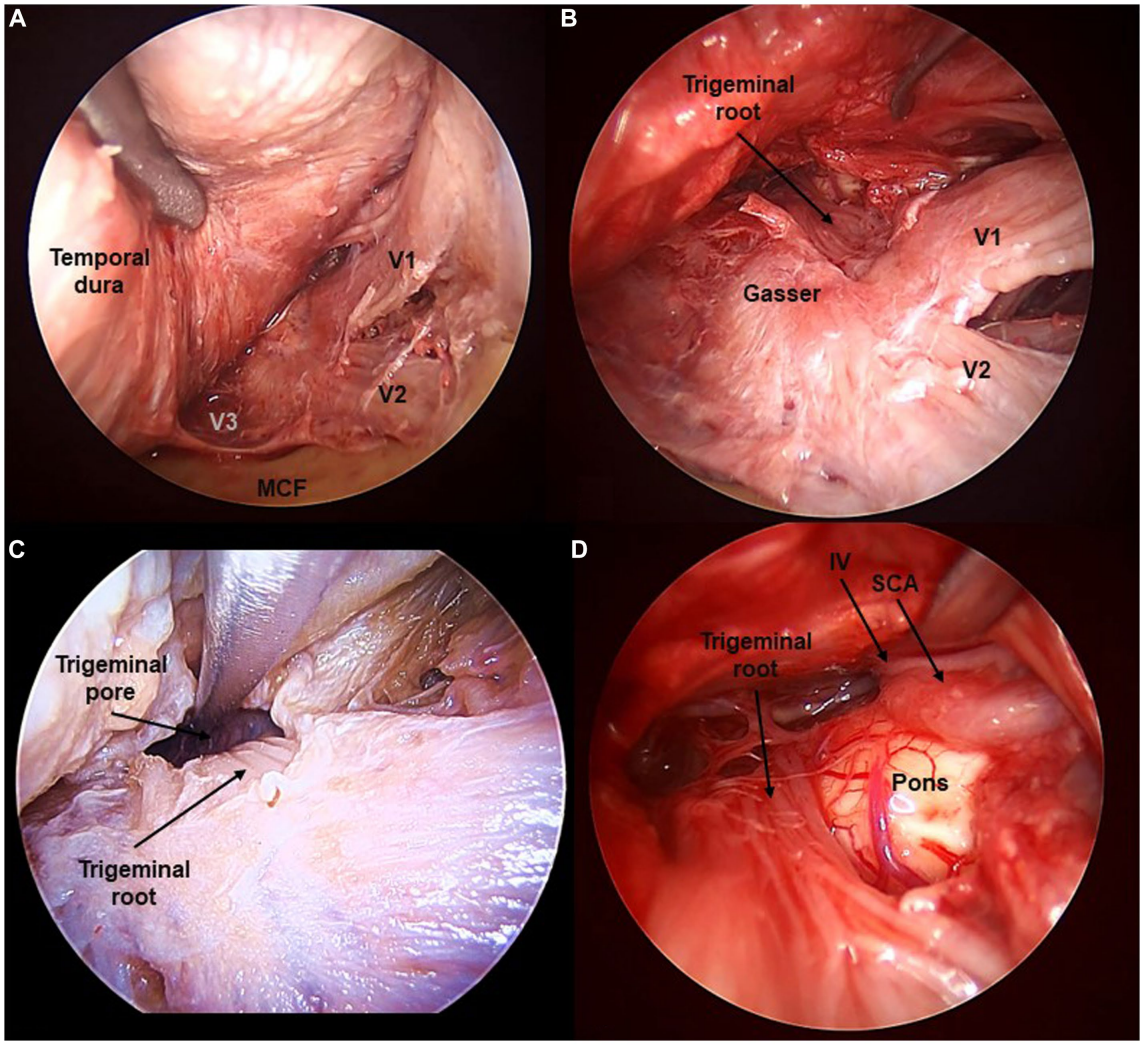
Fifth cranial nerve exposed from the transorbital perspective (Cadaveric dissection, left orbit). **(A)** The interdural peeling of the cavernous sinus, and extradural peeling of the middle cranial fossa (MCF) floor reveal the tree branches of the trigeminal nerve (V1, V2, V3). **(B)** Opening the dura in Meckel’s cave reveals the fibers of the trigeminal root. **(C)** Opening the trigeminal pore allows to follow the V nerve towards its course at the posterior fossa. **(D)** The trigeminal root is seen at its apparent origin in the ventrolateral pons. *SCA, Superior Cerebellar Artery*.

From the TO perspective, the cavernous course of the related cranial nerves can be inspected. V1 is seen traversing from the superior orbital fissure toward the Gasserian ganglion (GG). V2 is seen coming out from the pterygopalatine fossa through the foramen rotundum, and coursing posteriorly toward the GG. Meanwhile, V3 can only be seen if an extradural peeling of the middle fossa is performed or if the dura bands extending between V2 and V3 are cut. V3 runs within the middle fossa floor as it emerges from the infratemporal fossa through the foramen ovale to join the GG (Figure 4).

**Figure 5:**
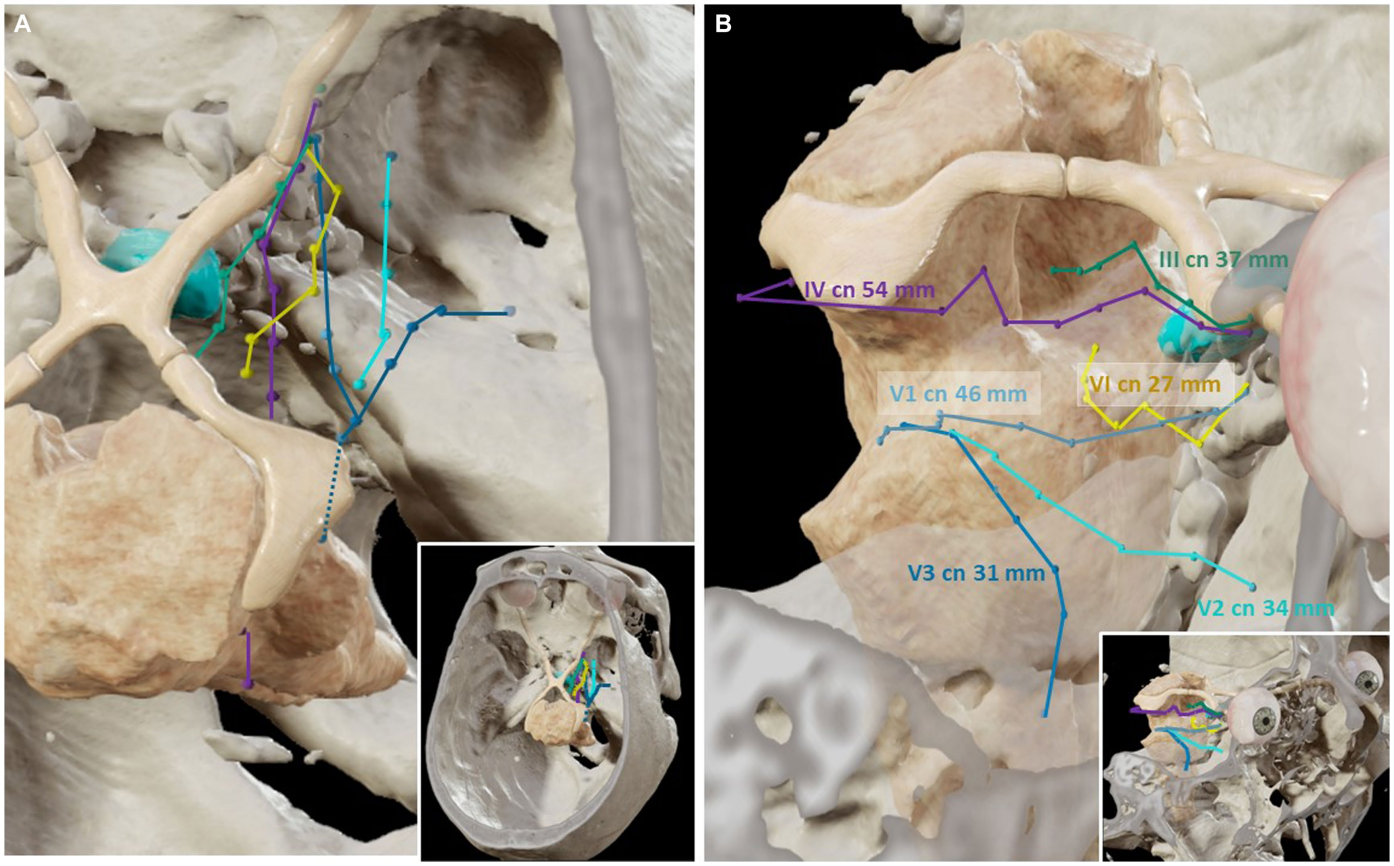
Three-dimensional reconstructions of the trajectories of the cranial nerves related to the cavernous sinus (Cadaveric specimens, Brainlab). **(A)** Superior view. The course of the cranial nerves related to the cavernous sinus has been plotted in the magnetic resonance imaging and computed tomography scans, according to the points obtained with the neuro-navigation system during the transorbital dissections. **(B)** The length of the course of each cranial nerve (cn) is shown.

The dura propria of the GG can be opened to enter a virtual arachnoid space known as Meckel’s cave, containing the trigeminal root fibers. The trigeminal root can be followed posteriorly, traversing the trigeminal porus toward the posterior fossa. Here, the trigeminal nerve crosses in intimate relation to the tentorial incisura and enters the anterior surface of the pons. Therefore, a complete exposure of the intracranial course of the trigeminal nerve is obtained with the TO approach. The average lengths of the trigeminal root and each of the branches seen transorbitally were 46 ± 2 mm for V1, 34 ± 3 mm for V2, and 31 ± 1 mm for V3 (Figure 5).

The III and IV cranial nerves share a similar trajectory within the lateral wall of the CS, within its superior aspect. They run from the anterior clinoid process toward Hakuba’s oculomotor triangle, located at the posterior part of the roof of the CS. Hakuba’s triangle, delimited by the petroclinoid and interclinoid ligaments, can be opened to unveil the course of these nerves within the basal cisterns. The III nerve is seen coursing between the posterior cerebral artery (PCA) and the superior cerebellar artery (SCA) before entering the midbrain at the interpeduncular fossa. The course of the III nerve is completely visible from the TO perspective, with a mean length of 37 ± 2 mm (Figures 1, 5). Meanwhile, the IV nerve courses above the SCA, within the ponto-mesencephalic sulcus, to reach its apparent origin in the posterior surface of the midbrain. The IV nerve cannot be completely followed to its apparent origin, and it is lost from the transorbital sight as it bends over the posterior margin of the cerebellar peduncle. The average length of the IV seen transorbital was 54 (±4) mm (Figures 2, 5).

Finally, to see the cavernous segment of the VI nerve, Mullan’s triangle needs to be opened as the nerve is purely intracavernous. Within the CS, the VI nerve courses in intimate contact with the ICA, in a rather inferior position. However, as the trajectory is followed deeper, the VI nerve adopts a rather lateral position toward the posterior wall of the CS. The VI nerve pierces the posterior wall of the CS toward the posterior cranial fossa. Concretely, the VI nerve passes under Grüber’s petrosphenoidal ligament, delimiting the so-called Dorello’s canal. The cisternal portion of the VI nerve can be seen after the anterior petrosectomy, although its low apparent origin may be obscured from the transorbital point of view. The average length of the VI seen was 27 ± 6 mm (Figures 3, 5).

## 4 Discussion

Since the earliest descriptions from Parkinson (Harris and Rhoton, 1976), the cavernous sinus (CS) has been well outlined from the transcranial (Harris and Rhoton, 1976; Taptas, 1982; Dolenc, 1983; Sekhar et al., 1987; Hakuba et al., 1989; Krisht et al., 1994; Kawase et al., 1996; Seoane et al., 2000; Yasuda et al., 2004, 2005) and the endoscopic endonasal (Knosp et al., 1991; Doglietto et al., 2009; Abuzayed et al., 2010; Erdogan et al., 2018; Fernandez-Miranda et al., 2018) perspectives. In this anatomical study, we unveil the CS from the endoscopic transorbital (TO) view, emphasizing the key landmarks to navigate this structure from this relatively novel standing point (Di Somma et al., 2018a). We provide illustrative and quantitative data regarding the course of the cranial nerves related to the CS, according to the TO approach extents and limitations.

The TO route offers straight access to the CS, particularly to its lateral wall. In fact, this lateral wall becomes the main surgical reference during the approach. It is composed of a two-layer dura fold as an extension of the dura lining the middle cranial fossa. At the inferolateral edge of the CS, right above the V2 branch, the inner (endosteal) and the outer (meningeal) layers separate, the former extends medially to form the medial sinus wall, which then bends at the roof of the CS to encircle the pituitary gland; the outer layer extends upwards and forms the lateral wall of the CS as the dura propria (Perneczky et al., 1988).

Through an interdural or an extradural dissection of the two dural layers, the CS can be exposed without transgression (Perneczky et al., 1988; Altay et al., 2012; Dallan et al., 2017). Commonly, both dissecting planes are combined to increase the exposure of the cavernous area. Moreover, the peeling of the CS is also intended to mobilize the temporal lobe laterally to gain extradural access to Meckel’s cave (Di Somma et al., 2020; Lima et al., 2020), the tentorial area (Lin et al., 2019; De Rosa et al., 2022), and the posterior cranial fossa (García-Pérez et al., 2022). Here, complementing the conventional TO approach with an anterior petrosectomy and the opening of the tentorium increases the view of the ventral pons and the cerebral peduncle area.

As first described by Umansky and Nathan (Umansky et al., 1994), the interdural dissection leaves a semi-transparent inner layer, which allows an exploration of the nerves coursing the lateral wall of the CS (III, IV, V1, and V2). The foramen rotundum, and indeed V2, will become key structures during the interdural peeling. For some authors, the lateral wall of the CS ends at the superior edge of V2 (Dolenc, 1983; Perneczky et al., 1988; Umansky et al., 1994; Seoane et al., 1998; Yasuda et al., 2005), yet in our experience with the TO perspective, V2 seems part of the lateral wall.

The lateral wall of the CS can be followed from the SOF (opened during the TO craniectomy), along the middle cranial fossa, and extending posteriorly to the petrous apex and petroclival fissure, inferiorly, and to the petroclinoid ligament and the trigeminal porus, superiorly. At the anterior margin of the CS, the III nerve is seen coursing lateral to the optic strut, at the lower margin of the anterior clinoid, as the most superior of all neural structures of the lateral wall of the CS. In the posterior part of the roof of the CS, the III nerve is seen entering the oculomotor triangle. This same triangle serves as an entrance point for the IV nerve, in a rather posterolateral position compared to the III. The IV nerve is also seen coursing the lateral wall of the CS, right below the III nerve, until it reaches the anterior clinoid process, where it bends medially and over the III to enter the orbit. The IV nerve is a landmark delimitating two anatomical triangles (the supratrochlear and infratrochlear) that serve as safe entry zones to the cavernous ICA and VI nerve from a TO perspective.

In addition, within this lateral wall, the ophthalmic division of the trigeminal nerve courses under the IV nerve. Posteriorly, the V1 branch joins the Gasserian ganglion, located just lateral to the CS within the Meckel’s cave. In turn, the maxillary division courses in the inferior margin of the CS lateral wall; at the inferomedial region of the middle fossa, V2 is seen traversing the foramen rotundum toward the pterygopalatine fossa. It is at this point of transition, over the V2 nerve, where the dura of the lateral wall unfolds into the two layers, separated by a trabecula of fibrous adhesions, which can be sharply divided to start the interdural dissection from a TO route.

Rather posteriorly and laterally within the middle fossa, the mandibular division of the trigeminal nerve is seen entering the foramen ovale into the infratemporal fossa. Of note, the V3 branch does not properly pertain to the lateral wall of the CS; therefore, exposing V3 requires an extradural peeling of the middle cranial fossa floor or, alternatively, cutting off the dura fibers that separate V3 from the lateral wall of the sinus during an interdural peeling.

Subject to decades of debate, the optimal surgical approach for accessing the CS is still unclear. The evolution of the transcranial approaches toward less invasive procedures also involved the CS. With the keyhole concept, the microscopic-based lateral TO (Altay et al., 2012; Matsuo et al., 2016; Chabot et al., 2017; Priddy et al., 2017) and the subtemporal (Taniguchi and Perneczky, 1997; Pichierri et al., 2010) approaches provided more conservative anterolateral and lateral routes to the CS and Meckel’s cave. However, the main limitation of these keyhole strategies is the narrow surgical corridor, causing the depth of the surgical field severe limitations of visualization, surgical freedom, and instruments maneuverability. The ultimate advance in the minimally invasive trend was the combination of these small ports with the endoscopic armamentarium (Perneczky and Fries, 1998), in which the TO route has its fundament.

The endoscopic extended endonasal approach can provide access to the anterior and middle walls of the CS, by means of an ethmoido-pterygo-sphenoidal removal (Cappabianca et al., 1998; Cavallo et al., 2005; Doglietto et al., 2009). If drilling is extended laterally to the parasellar ICA, the lateral wall of the CS can also be exposed (Abuzayed et al., 2010) by retracting the ICA and the VI cranial nerve medially. From the endonasal perspective, an anteromedial corridor can be opened to reach the lateral CS and Meckel’s cave (Kassam et al., 2009). Arguably, exposure of these lateral components of the CS is more feasible through the TO route as it avoids an extensive exposure and manipulation of the ICA (Alfieri and Jho, 2001; Zoia et al., 2024). Conversely, to access the medial CS the endonasal corridor is superior to the TO (Alfieri and Jho, 2001; Cavallo et al., 2005; Doglietto et al., 2009; Abuzayed et al., 2010). Still, these potential advantages mostly apply to pituitary adenomas extending to the CS (Yokoyama et al., 2001) and not to other tumoral or vascular lesions. Plus, a major drawback of the anterior corridor is dealing with a hypothetical injury to the ICA, which is the main boundary in the lateral extension of the surgical corridor.

Complementing the limitations of the endonasal technique, the endoscopic lateral TO approach affords a direct route to the lateral wall of the CS (Altay et al., 2012; Bly et al., 2014; Matsuo et al., 2016; Dallan et al., 2017). Compared to the subtemporal and orbitozygomatic approaches, the need for temporal lobe retraction seems to be remarkably reduced, but at the expense of less surgical freedom in the posterior region of the CS, particularly in the vertical plane (Lima et al., 2020). However, this limitation could be reduced by transient the removal of the orbital rim. Alternatively or additionally, the use of a multi-portal strategy where two or three surgical corridors are exploited simultaneously may improve the visualization and range of motion in deep-seated areas of the skull base (Corvino et al., 2023; Zoia et al., 2023).

This study provides comprehensive data on the anatomy of the lateral wall and internal components of the CS from a TO perspective. It describes the course of the cranial nerves related to the CS, to allow safe navigation of this complex structure, which is indeed in the culminating learning process of the TO surgery (Di Somma et al., 2022). Still, some limitations inherent to the use of cadaveric specimens prevent direct extrapolation from these data to the surgical field. Further studies are warranted.

## Data availability statement

The original contributions presented in the study are included in the article/Supplementary material, further inquiries can be directed to the corresponding author.

## Ethics statement

The studies involving humans were approved by IRB of the University of Barcelona (Barcelona, Spain). The studies were conducted in accordance with the local legislation and institutional requirements. The ethics committee/institutional review board waived the requirement of written informed consent for participation from the participants or the participants’ legal guardians/next of kin because Cadaveric specimens, they gave their informed consent for biomedical investigation, but not specifically for this project.

## Author contributions

AM: Writing – original draft. MC: Writing – review & editing. RT: Writing – review & editing. RM: Data curation, Investigation, Writing – review & editing. JT: Writing – review & editing. JE: Writing – review & editing. AS: Writing – review & editing. AP-G: Writing – review & editing.
